# Repeat and single dose administration of gadodiamide to rats to investigate concentration and location of gadolinium and the cell ultrastructure

**DOI:** 10.1038/s41598-021-93147-2

**Published:** 2021-07-06

**Authors:** Julie Davies, Michael Marino, Adrian P. L. Smith, Janell M. Crowder, Michael Larsen, Lisa Lowery, Jason Castle, Mark G. Hibberd, Paul M. Evans

**Affiliations:** 1 GE Healthcare, Pollards Wood, Nightingales lane, Chalfont St. Giles, UK; 2 GE Global Research Centre, 1 Research Circle, Niskayuna, NY USA; 3 GE Healthcare, Life Sciences, Marlborough, MA USA

**Keywords:** Medical imaging, Electron microscopy, Preclinical research

## Abstract

Gadolinium based contrast agents (GBCA) are used to image patients using magnetic resonance (MR) imaging. In recent years, there has been controversy around gadolinium retention after GBCA administration. We sought to evaluate the potential toxicity of gadolinium in the rat brain up to 1-year after repeated gadodiamide dosing and tissue retention kinetics after a single administration. Histopathological and ultrastructural transmission electron microscopy (TEM) analysis revealed no findings in rats administered a cumulative dose of 12 mmol/kg. TEM-energy dispersive X-ray spectroscopy (TEM-EDS) localization of gadolinium in the deep cerebellar nuclei showed ~ 100 nm electron-dense foci in the basal lamina of the vasculature. Laser ablation-ICP-MS (LA-ICP-MS) showed diffuse gadolinium throughout the brain but concentrated in perivascular foci of the DCN and globus pallidus with no observable tissue injury or ultrastructural changes. A single dose of gadodiamide (0.6 mmol/kg) resulted in rapid cerebrospinal fluid (CSF) and blood clearance. Twenty-weeks post administration gadolinium concentrations in brain regions was reduced by 16–72-fold and in the kidney (210-fold), testes (194-fold) skin (44-fold), liver (42-fold), femur (6-fold) and lung (64-fold). Our findings suggest that gadolinium does not lead to histopathological or ultrastructural changes in the brain and demonstrate in detail the kinetics of a human equivalent dose over time in a pre-clinical model.

## Introduction

Gadolinium-based contrast agents (GBCAs) are frequently used to enhance magnetic resonance (MR) imaging and have been utilised in the clinical setting for more than 30 years. In recent years hyperintensity on unenhanced T1-weighted MR images in specific brain regions such as the dentate nucleus have been identified in patients with a history of GBCA administration^1–3^. There is a correlation between MR hyperintensity and brain gadolinium in both patients^3,4^ and in animal studies following GBCA administration^5–9^. From these animal studies it has been demonstrated that gadolinium retained in the rat brain is apparent for at least 1 year^7,10–13^ after multiple GBCA dose administrations. T1-weighted image hyperintensity derives from gadolinium present as the intact GBCA or bound to macromolecules. As gadolinium must be in solution to relax water protons, insoluble gadolinium is unlikely to contribute to hyperintensity and therefore MR hyperintensity may underestimate the actual gadolinium presence in the brain. Whilst these repeat dose animal studies are informative, they do not represent a likely clinical scenario. Retention is mostly focused in the deep cerebellar nuclei (DCN), is present after administration of both linear and macrocyclic agents^13,14^ and is not associated with any evidence of tissue injury. In addition to an absence of histopathological sequalae, multiple or supraclinical doses of GBCAs in rats have no effect on biochemical parameters or in a multitude of behavioural assessments^15,16^. Pups of pregnant mice administered multiple doses of either gadodiamide or gadoterate (13 times the human equivalent dose from gestational days 15–19) displayed impairment in rotarod and object location tests^17^. The significant behavioural differences reported in this study did not correlate to either the level or presence of gadolinium in the rat brain and may be related to other aspects of administering large amounts of GBCA during gestation using a dosing regimen that has little relevance to clinical use.

A recent study^18^ which assessed gadolinium levels in a limited number of tissues after high, repeated GBCA administration to rats, does not provide retention kinetics after a single human equivalent dose, which is more suitable a comparison to the clinical setting. Our study sought to understand not only the long-term retention kinetics following a cumulative dose, but importantly gadolinium retention after a single human equivalent dose, more relevant to the clinical use of GBCAs. There is a need to better understand the kinetics after single dose administration to comprehend the clearance of gadolinium from the system over a prolonged period as well as organ specific distribution. Given that there is concern over the longer-term retention of gadolinium, discreet regions of the brain and other important organs were assessed to more fully characterize the pharmacokinetics of a single GBCA administration and histopathology was performed on the brains of the high dose animals.

## Materials and methods

The repeat dose part of this study was performed independently of GE Healthcare at the GE Global Research Center and was approved by the GE Global Research Center Institutional Animal Care and Use Committee. The single dose gadodiamide kinetics portion of this study was performed independently at QPS in the Netherlands and Austria, the vivarium at QPS is a fully Association for Assessment and Accreditation of Laboratory Animal Care (AAALAC) accredited facility. All procedures in this study complied with the Animal Care and Welfare Committee (ACWC). Animals were maintained according to the animal welfare regulations of the Ministry of Science of the Austrian government for work completed at QPS.

All experiments were conducted in compliance with ARRIVE guidelines. Long term studies were financially supported by GE Healthcare and performed by GE Global Research Centre. The gadolinium kinetics portion of the study was supported by a financial contribution from GE healthcare but conducted independently by QPS.

### Animals

Male Sprague Dawley rats aged 6 weeks were purchased from Envigo and were housed with a maximum of 4 rats per cage at approximately 21 °C and 40–70% humidity with food and water ad libitum on a 12-h light–dark cycle. Animals were acclimated for 7 days prior to initiation of study and checked for their health status. Only animals in good health were included in the study and rats were randomly assigned to groups. Throughout the duration of the study animals were observed once daily for clinical signs and weights were recorded prior to the start, on the treatment day and weekly thereafter. Rats underwent repeat dose administration as previously described^11^. In brief, rats (n = 6 per group) were administered intravenous gadodiamide (GE Healthcare, Chalfont St Giles, UK) in a lateral tail vein at a dose of 0.6 mmol/kg, 4 times a week for 5 weeks (a cumulative dose of 12 mmol/kg) or saline for the control group. Animals were anesthetised with pentobarbital and transcardically perfused with neutral buffered formalin (n = 3) or Karnovsky’s fixative (n = 3) at 1-, 20- or 50-weeks post-dosing. Brains were harvested and bisected with one hemisphere used for ICP-MS and the other for histopathology, TEM, and LA-ICP-MS. For single dose gadodiamide kinetics, rats (n = 3 per timepoint) were administered 0.6 mmol/kg of gadodiamide in a lateral tail vein. At determined time points (1 h, 1 day, 3 days, 1 week, 2 weeks, 3 weeks, 4  weeks, 6 weeks, 8 weeks, 10 weeks, 15 weeks or 20 weeks) animals were anesthetised using pentobarbital and CSF was collected from the cisterna magna and blood by cardiac puncture. Animals were transcardially perfused and the cerebellum was removed, the cerebrum was then hemisected into cortex, subcortex (striatum and thalamus), hippocampus and the remaining undissected regions of the brain were pooled to form a sample we have termed rest of the brain. The left kidney, left liver lobe, left lung lobe, femur bones, testes and skin (shaved 1 × 1 cm square) were also collected. All samples were stored at − 80 °C and analysis of samples was performed by ICP-MS. There were no adverse events or unexpected deaths. There were no significant differences in body weight increase between groups at any point and animals had general good health.

### Histopathology

Histopathology was performed by an independent pathologist as previously described^11^ for animals treated with a cumulative gadodiamide (12 mmol/kg) or saline control at 1-, 20 and 50-weeks post dosing (n = 6 per group). In brief, perfusion-fixed tissues were cut into 1 mm sections at three standard levels (coronal tissue blocks corresponding to levels 1–3 as defined by; (1) cerebrum at the optic chiasm, (2) cerebrum at the base of the posterior hypothalamus (3) mid-cerebellum and medulla oblongata), fixed overnight in NBF or Karnovsky’s fixative, and then processed into paraffin blocks in a tissue processor. Paraffin blocks were shipped to Pathology Associates International, a division of Charles River Laboratories (Maryland, USA), where they were sectioned, stained, and evaluated by a toxicologic histopathologist using light microscopy (Olympus BX45) and assessing morphology and histopathological changes. Histopathologists were initially blinded to treatment group but not to the study treatment agent.

### Transmission electron microscopy ultrastructural analysis

TEM was performed for ultrastructural analysis. The 1 mm coronal sections taken from the mid-cerebellum and medulla oblongata of Karnovsky’s-perfused rats treated with a cumulative dose (12 mmol/kg) of gadodiamide 20- and 50-weeks post dosing (n = 3 per group) were fixed overnight in fresh fixative. The sections were then further dissected to yield notched blocks of dimensions 1 × 1 × 2 mm, centered on the deep cerebellar nuclei, with the notch providing orientation. These small blocks were then transferred to fresh Cacodylate buffer (0.1 M, pH 7.4) and shipped to Pathology Associates International, a division of Charles River Laboratories (Maryland, USA), where they were stained with both osmium and uranium stains, dehydrated, embedded in resin, and sectioned for analysis by TEM. Images collected by TEM were evaluated by a toxicologic histopathologist specializing in TEM blinded to treatment group.

### Transmission electron microscopy and energy-dispersion spectrometry

TEM–EDS was performed to identify gadolinium foci at the microscopic level in animals treated with a cumulative dose (12 mmol/kg) of gadodiamide at 50-week post dosing to assess gadolinium localization in the DCN (n = 3). For this analysis blocks were collected and dissected in a similar fashion as for ultrastructural analysis, but were not stained, and were instead dehydrated and embedded in resin directly. Sections (100 nm thick) were subsequently stained with uranyl acetate (2% w/v in H_2_O) and analyzed on a FEI Tecnai Osiris S/TEM with an X-FEG high-brightness Schottky field emission source operated at 200 kV accelerating voltage. The microscope was operated in the scanning mode with a sub-nanometer electron beam diameter and a point-to-point image resolution of less than 0.2 nm. Spectral images were obtained with a Super-X EDS system with four integrated silicon drift detectors with an X-ray solid angle of 0.9 mrads and a windowless detector that allows the detection of all elements down to and including boron. Gadolinium detection by EDS is confounded by osmium, which precludes the osmium fixation that is required for ultrastructural analysis, so only one treatment group—1-week post-dosing—was analyzed by this method.

### Laser ablation with inductively coupled plasma mass spectroscopy

LA-ICP-MS was performed to asses elemental bioimaging of gadolinium retention in animals treated with a cumulative dose (12 mmol/kg) of gadodiamide or saline controls (n = 3 per group) at 1-, 20- or 50-week post dosing at three standard levels (coronal tissue blocks corresponding to levels 1–3 as defined by; (1) cerebrum at the optic chiasm, (2) cerebrum at the base of the posterior hypothalamus, (3) mid-cerebellum and medulla oblongata). This was performed using a laser ablation system (213 nm LSX-213, Cetac Technologies, Omaha, NE) coupled to a Perkin Elmer (Waltham, MA) Elan DRC II ICP-MS. Brain sections were taken from each of the levels as described above at 1, 20 and 50 weeks post 12 mmol/kg gadodiamide injection. Sections 50 μm thick were cut directly from paraffin blocks, were ablated with a multiline raster sequence, and 2D elemental distribution maps were generated with an in-house mapping program based on MATLAB software (MathWorks, Natick, MA). Maps of high spatial resolution were generated using a 50 μm spot size, 20 Hz laser frequency, 80% laser power, 50 μm/s raster rate, 20 μm spacing between raster lines and 40 s gas blank delay between laser lines. Each tissue was mapped in two sections and the maps joined to create a single map image. Maps of low spatial resolution were generated using a 145 μm diameter square beam, 10 Hz laser frequency, 50% laser power, 50 μm/s raster rate, 20 μm spacing between raster lines and 40 s gas blank delay between laser lines. Each tissue was mapped in a single section. Complete ablation of the tissue was achieved under both laser conditions. The ICP-MS was operated at 1100 W RF power, 15.0 L/min Ar plasma gas flow, 1.2 L/min Ar auxiliary gas flow, and 1.2 L/min Ar carrier gas flow through the laser ablation cell. The sampler and skimmer cones were nickel. The dwell time was 47 ms per isotope *m/z* monitored (^13^C, ^24^Mg, ^27^Al, ^31^P, ^33^S, ^34^S, ^158^Gd, ^160^Gd, ^232^Th) resulting in 0.50 s read time per replicate. Isotopes ^158^Gd and ^34^S were selected for creation of the gadolinium and sulphur maps. NIST SRM 612 trace elements in glass were ablated before and after each brain map to verify the performance of the laser ablation and ICP-MS systems.

### Inductively coupled plasma mass spectroscopy

ICP-MS analysis to measure gadolinium concentration was performed on tissue, blood and CSF samples of animals (n = 3 per timepoint) treated with a human equivalent dose at various timepoints (1 h, 1 day, 3 days, 1 week, 2 weeks, 3 weeks, 4 weeks, 6 weeks, 8 weeks, 10 weeks, 15 weeks or 20 weeks) and in the left hemisphere of rats administered a cumulative (12 mmol/kg) gadodiamide dose or a saline control at 1, 20, or 50 weeks post dosing (n = 6 per group). To measure concentrations of gadolinium in whole blood and CSF samples; 50 µL of nitric acid with 0.01% Tween-20 and 1500 µL of 1 µg/L terbium (Sigma-Aldrich) was added to each sample. For tissue digestion, to each sample a 20 mM solution of pentetic acid in 65% nitric acid with terbium (1 mg/mL) was added. Samples were then incubated at 100 °C in a DigiPREP (SCP science) for 70 min after which an equal volume of hydrogen peroxide was added and incubated for 20 min at 100 °C. Samples were then processed for ICP-MS measurement by addition of 50 µL of calibration solution and 1050 µL of 1% nitric acid. Gadolinium was measured using the continuous flow analysis method (ALS autosampler). Gadolinium concentrations were recorded as nmol/g or %ID/g for tissue samples and nmol/mL or %ID/mL for whole blood and CSF samples.

### Statistical analysis

Data and statistical analysis were performed using Prism 8.0.2 (GraphPad Software). Data were tested for normal distribution and homogeneity of variance using a Shapiro–Wilk and Levene’s test, respectively. Statistical analyses were performed using a one-way ANOVA followed by a Dunnett’s post-hoc test. Significance was assigned to differences of p values less than or equal to 0.05.

## Results

### Trace levels of gadolinium persisted for a year post-injection without apparent tissue injury after repeated gadodiamide administration

**Figure 1:**
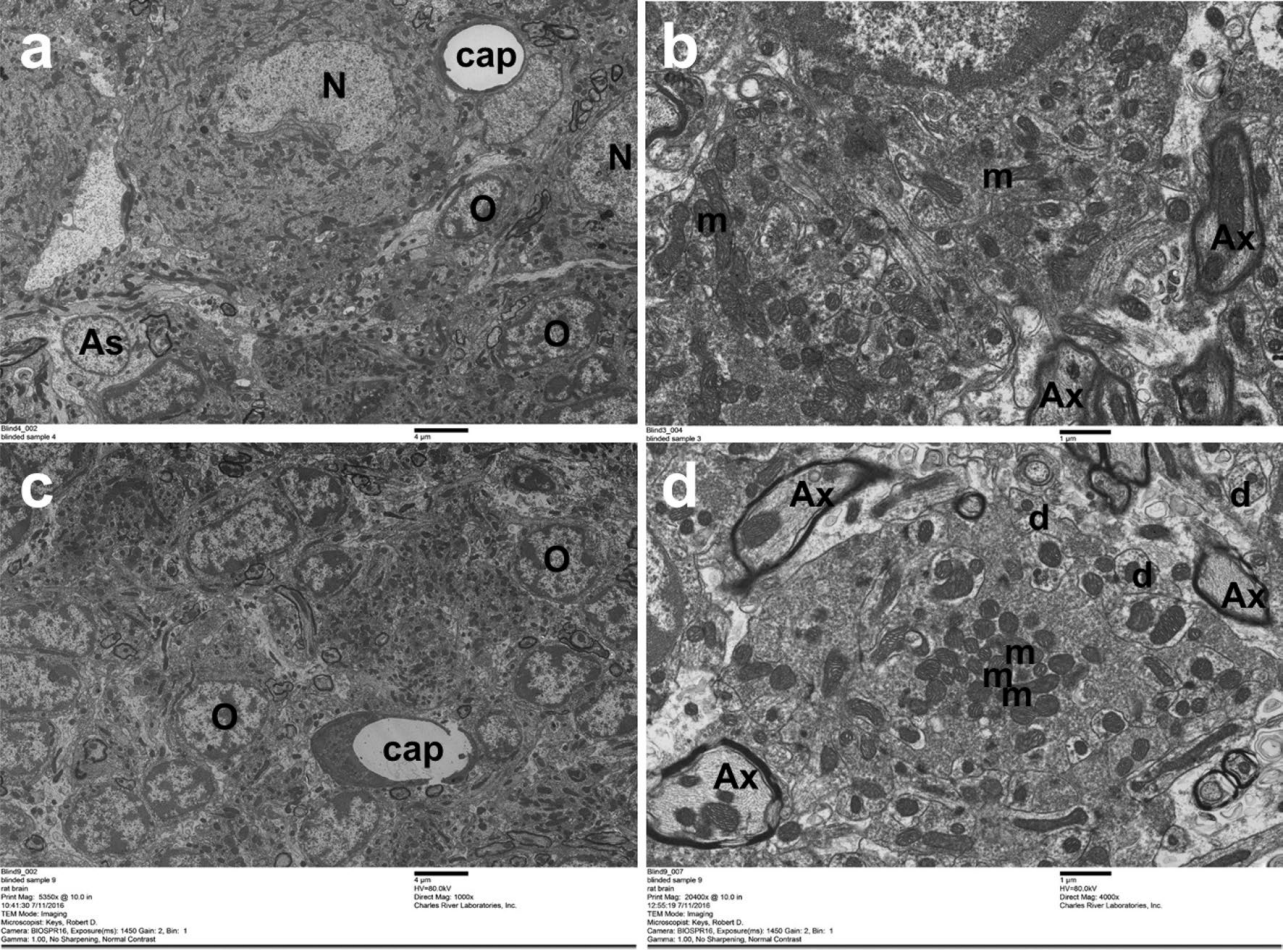
Normal ultrastructure in the deep cerebellar nuclei after repeated doses of gadodiamide. Figures show ultrastructural resolution in the deep cerebellar nuclei after treatment with (**a**,**b**) gadodiamide or (**c**,**d**) saline control group. (**a**,**c**) Low power magnification ×1000, scale bar 4 µm. (**b**,**d**) High power magnification ×4000, scale bar 1 µm. *As* astrocyte nucleus, *Ax* axon, *cap* capillary, *d* dendrite, *m* mitochondria, *N* neuronal nucleus, *O* oligodendrocyte nucleus. Images are representative (n = 3 per group).

The gadolinium concentration in the whole brain at 50 weeks post-injection was not statistically different from that at 20 weeks (1.56 ± 0.19 vs 1.38 ± 0.11 nmol/g respectively, p < 0.34; Table 1) but there was a significant reduction between 1 and 20 weeks post-injection (2.59 ± 0.30 vs 1.38 ± 0.11 nmol/g, respectively, p < 0.001; Table 1). Long-term trace levels of gadolinium in the rat brain did not lead to tissue injury. In line with the previously reported study^11^, three anatomic levels in the 1-year treatment group were evaluated by an independent toxicologic pathologist with no morphological changes reported. The majority of cerebellar images evaluated were considered within normal limits. In one control animal, mild axonal degeneration was noted, as was mild lipid accumulation in an unidentified cell type adjacent to a capillary but given the low grade and limited distribution of these findings, they were considered incidental. Isolated endothelial degeneration was noted in one vessel of one animal in each of the treatment groups and were considered incidental findings. Further to the assessment by light microscopy, no treatment-related effects were apparent by TEM in either the 20- and 50-week group, nor were there any differences in morphology of neurons, astrocytes, oligodendrocytes, microglia, endothelium or pericytes (Fig. 1).

**Table 1:** ICP-MS measurement of brain gadolinium in whole brain in rats administered a cumulative dose of 12 mmol/kg over 5 weeks (n = 6 per group).

Time post-dosing (weeks)	Mean (nmol/g)	SD	Significance
1	2.59	0.30	NS
20	1.38	0.11	p < 0.001 vs 1 week
50	1.56	0.19	p < 0.34 vs 20 week

*Ns* not significant. Statistical analyses using a one-way ANOVA with a Dunnet’s post-hoc test.

### Gadolinium is present in most brain regions but is focally concentrated in the deep cerebellar nuclei and confined to the vascular wall

**Figure 2:**
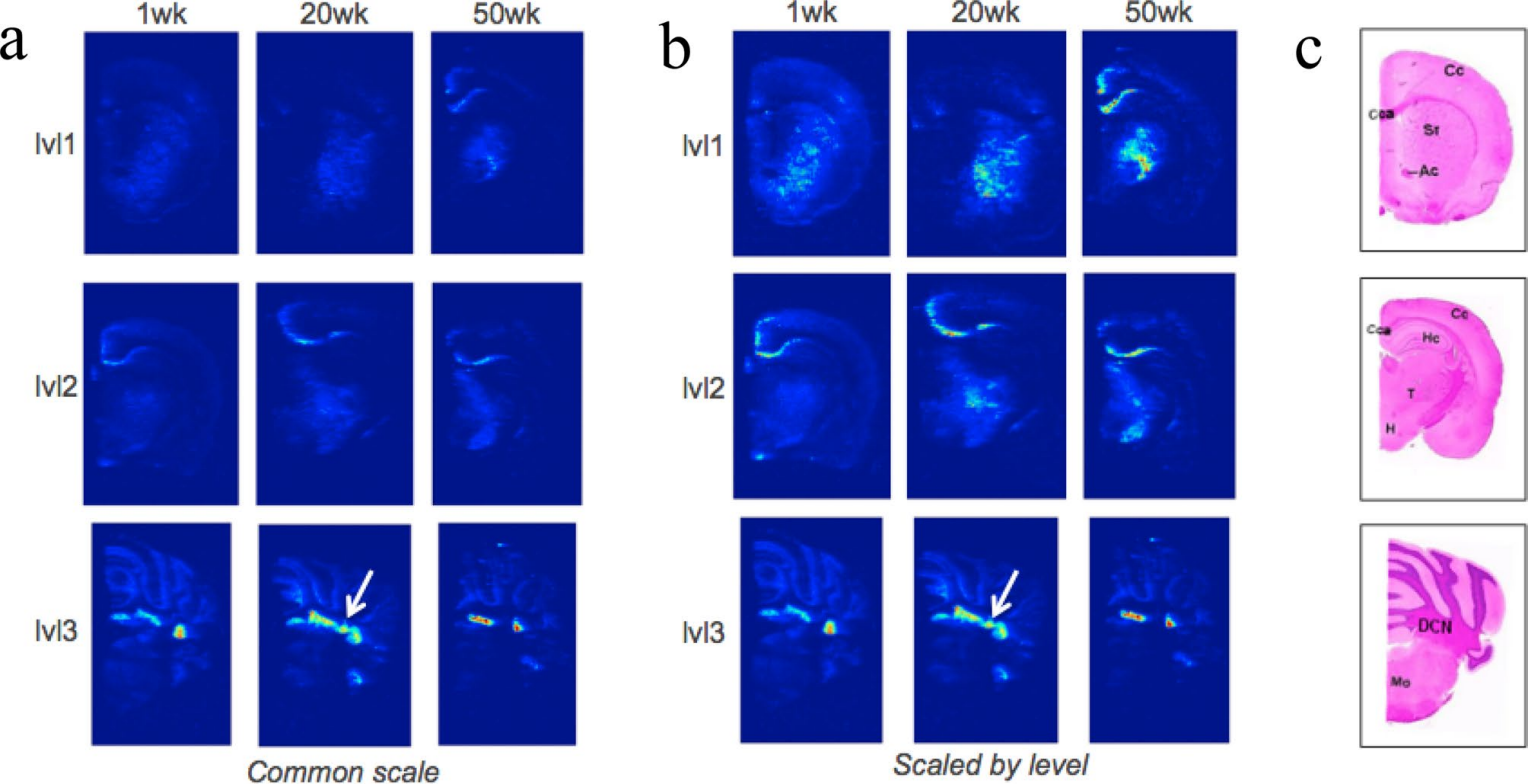
Gadolinium distribution in the rat brain after a cumulative gadodiamide dose. Gadolinium distribution is consistent over a 1-year period following a cumulative dose of 12 mmol/kg over 5 weeks (n = 3 per group), with the highest concentration in the DCN. Localization of gadolinium across the brain measured by LA-ICP-MS. (**a**) representative images from each brain level and each treatment group on a common scale—the highest concentration of gadolinium is observed in the DCN in level 3 (arrow, bottom row). (**b**) The same images with each level exhibited on a different scale to highlight areas of gadolinium in each. (**c**) Example H&E images of the same levels with major features marked (levels are defined as (1) cerebrum at the optic chiasm, (2) cerebrum at the base of the posterior hypothalamus (3) mid-cerebellum and medulla oblongata). *Ac* anterior commissure, *Cc* cerebral cortex, *Cca* corpus callosum, *DCN* deep cerebellar nuclei, *Hc* hippocampus, *H* hypothalamus, *Mo* medulla oblongata, *Sr* striatum, *T* thalamus.

Mesoscale localization of gadolinium by LA-ICP-MS (Fig. 2) revealed diffuse distribution across most brain regions but with focal concentration in the DCN and the cerebellar granular cell layer. Comparisons of all three tissue levels from 1, 20, and 50 weeks post-injection revealed similar distribution patterns. In level 1 (cerebrum at the optic chasm), gadolinium was apparent in subcortical structures which appear to be in the region of the caudoputamen/striatum which includes the globus pallidus. Periventricular lateral focal concentrations were also seen in some animals. Similarly, in level 2 (cerebrum at the base of the posterior hypothalamus), diffuse gadolinium is seen in subcortical structures which include the thalamus, midbrain, and hypothalamus with lateral periventricular focal concentrations in some animals. In level 3 (mid-cerebellum and medulla oblongata), focal concentration in the DCN was in excess of that observed anywhere else in the brain.

**Figure 3:**
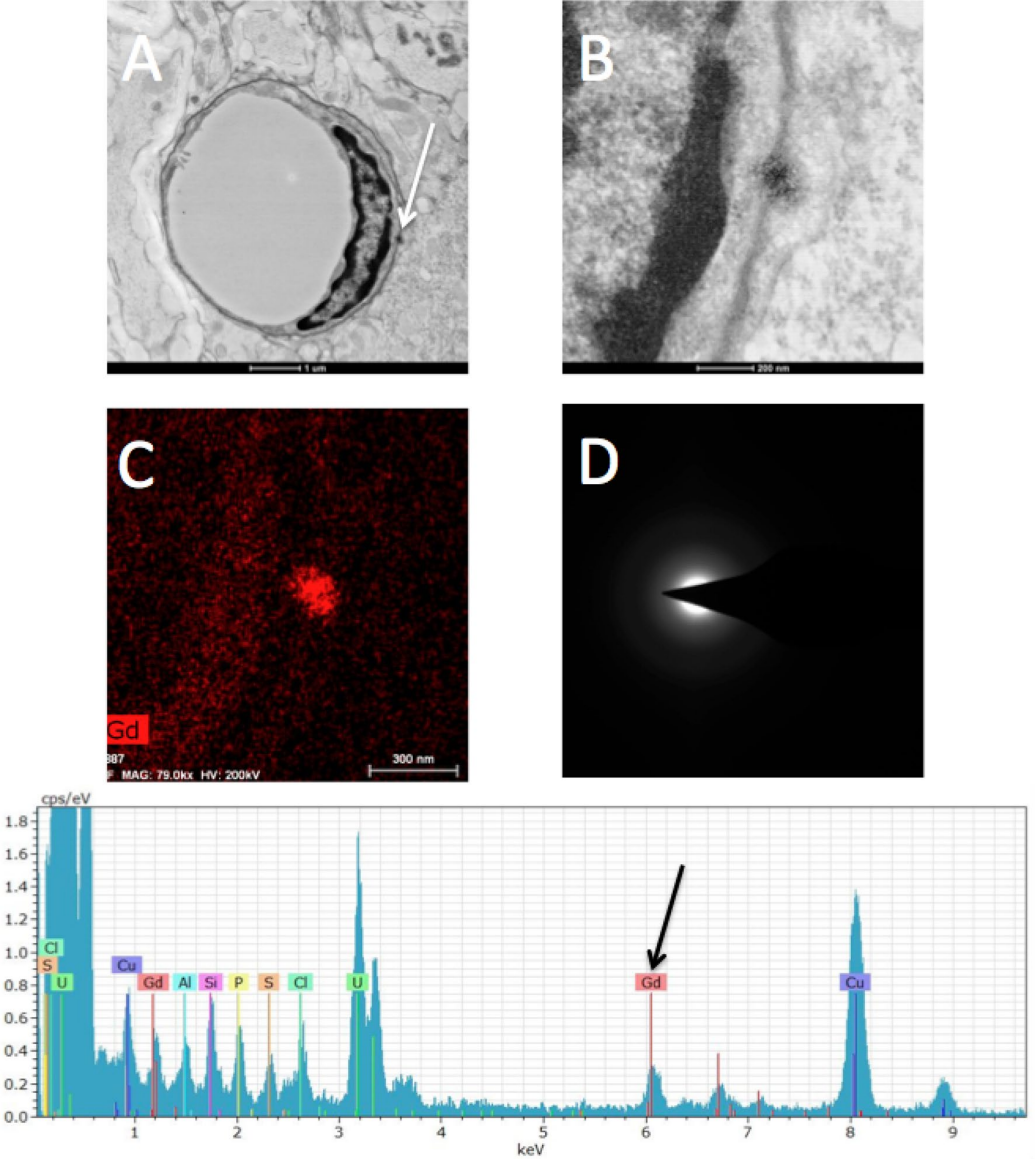
Localization of gadolinium after repeated gadodiamide administration. Most gadolinium observed in the DCN is in perivascular foci (n = 3 per group). Representative TEM with EDS of the DCN 1-year post dosing in a rat treated with 12 mmol/kg gadodiamide over 5 weeks. (**A**) low-magnification (×14,000) TEM image of a capillary with an electron dense focus indicated by white arrow. (**B**) high-magnification (×79,000) TEM image of the same focus. (**C**) High magnification TEM image filtered to show potential gadolinium-positive foci. (**D**) Diffraction image from an electron dense focus showing no diffraction—this implies an amorphous, non-crystalline structure. Bottom—spectrum obtained from the center of the focus shown in (**A**–**C**) above. A definitive gadolinium spectrum is observed.

At the microscopic scale TEM-EDS confirmed gadolinium foci in the vasculature of the DCN, predominantly on the luminal side of the blood brain barrier (BBB) (Fig. 3). After viewing approximately 200 randomly selected fields of view with no preference given to specific structures, 12 vascular foci were observed and only one non-vascular focus was observed. Vascular foci were consistently at the interface between the endothelial cell and the basal lamina. The gadolinium positive foci were ~ 100 nm spherical electron-dense foci that exhibited spectral characteristics consistent with gadolinium. The lone focus in the neuropil was not associated with any specific structure but appeared to be extracellular. X-ray diffraction analysis confirmed foci were amorphous, seemingly ruling out crystalline deposits of inorganic salts.

### Gadolinium is detected in all tissues post a single administration but shows time dependent reductions

**Figure 4:**
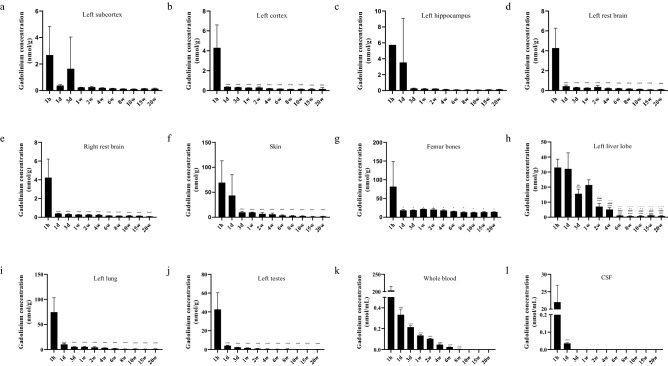
Gadolinium concentrations in tissues after a single gadodiamide administration. Gadolinium concentrations in whole cerebellum (**a**) Left subcortex, (**b**) left cortex, (**c**) left hippocampus, (**d**) left rest brain, (**e**) right restbrain, (**f**) skin, (**g**) femur bones, (**h**) left liver lobe, (**i**) left lung, (**j**) left testes, (**k**) whole blood and (**l**) CSF after rats were administered 0.6 mmol/kg gadodiamide. Measured gadolinium was BLOQ after 10 weeks in the whole blood and 3 days in the CSF. Graphs show mean ± SD (n = 3 per time point). Statistical analyses using a one-way ANOVA with a Dunnet’s post-hoc test, p ≤ 0.05*, < 0.01**^/##/++/~~^, < 0.001***/^~~~, ^< 0.0001****/^~~~~^. The significance level of each comparison is indicated for each column when compared to 1 h (*), 1 day (#), 3 days (+) or 1 week (~) time points.

**Figure 5:**
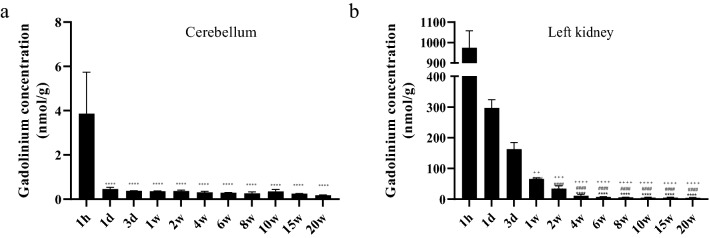
Gadolinium concentrations in the whole cerebellum and left kidney after a single gadodiamide administration. (**a**) Gadolinium concentrations in whole cerebellum shows initial rapid clearance and a slower reduction in gadolinium over 20 weeks after a 0.6 mmol/kg administration in rats. (**b**) Kidney gadolinium concentrations demonstrate triphasic clearance with initial rapid clearance up to one-day, a slower clearance up to 2 weeks and a plateau up to 20 weeks after a single administration of gadodiamide. Graphs show mean ± SD (n = 3 per time point). Statistical analyses using a one-way ANOVA with a Dunnet’s post-hoc test, p ≤ 0.01^++^, < 0.001^+++^, < 0.0001****^/####/++++^. The significance level of each comparison is indicated for each column when compared to 1 h (*), 1 day (#) or 3 days ( +) time points.

Given that there was a significant reduction in tissue gadolinium between 1 and 20 weeks postdosing, but no significant difference between 20 and 50 weeks, we wanted to better understand the kinetics with a more focused analysis up to 20 weeks. ICP-MS analysis of total gadolinium in the whole blood of animals administered gadodiamide revealed an exponential decrease from 203.8 ± 11.6 µg/L, 1-h post-injection to 0.33 ± 0.05 µg/L 1-day post-injection, which continued to decrease until 8 weeks, after which gadolinium was below the limit of quantification (BLOQ) (Tables 2, 3, Fig. 4). Gadolinium concentration in the CSF followed similar pharmacokinetics and was initially high (22.9 ± 4.9 µg/L) with a reduction after 1 day (0.03 ± 0.004 µg/L) and was BLOQ after 3 days. Gadolinium 1-h post-injection in the brain regions sampled, namely; whole cerebellum (3.9 ± 1.9 nmol/g), left cortex (4.3 ± 2.3 nmol/g), left subcortex (2.7 ± 2.2 nmol/g), left hippocampus (5.7 nmol/g), left restbrain (4.3 ± 2 nmol/g) and right rest brain (4.23 ± 2 nmol/g) had a peak concentration which decreased between 7 and 18 fold, 1-day post-injection (Figs. 4, 5). From 1 day until 20 weeks post-dosing there was a reduction in all these regions and by 20 weeks gadolinium levels were significantly lower but remained detectable. The amount of gadolinium as a percentage of the injected dose (ID) for the brain regions analysed 1-h post-injection was lowest in the subcortex (0.0004 ± 0.0005% ID/g) and highest in the left restbrain (0.0009 ± 0.0004% ID/g). Twenty-weeks post-dosing the lowest brain gadolinium measured was 0.00002 ± 0.000002% ID/g in the hippocampus and highest in the cerebellum (0.00004 ± 0.000004% ID/g). Gadolinium concentrations 1-h post-injection in the skin (68.4 ± 43.7 nmol/g, 0.015% ± 0.002% ID/g), femur bones (81.7 ± 67.5 nmol/g, 0.02 ± 0.01% ID/g), left kidney (974.6 ± 82.9 nmol/g, 0.2 ± 0.01% ID/g), left liver lobe (33 ± 5.6 nmol/g, 0.007 ± 0.001% ID/g), testes (42.6 ± 17.8 nmol/g, 0.009 ± 0.004% ID/g) and left lung lobe (74.5 ± 28.7 nmol/g, 0.01% ± 0.005% ID/g) were all significantly higher than in the brain at the same time point (Figs. 4, 5). Almost all these tissues (except the femur and kidney) displayed first order kinetics in the reduction of gadolinium up to 20 weeks, where they were significantly lower.

**Table 2:** Gadolinium concentrations as measured by ICP-MS after a single dose of gadodiamide (0.6 mmol/kg) in blood, CSF and tissues (n = 3 per group), ± S.D.

Tissue	Timepoint										
	1 h	1 day	3 day	1 week	2 weeks	4 weeks	6 weeks	8 weeks	10 weeks	15 weeks	20 weeks
Whole blood (nmol/mL)	203.78 ± 11.60	0.33 ± 0.05	0.21 ± 0.01	0.13 ± 0.007	0.10 ± 0.005	0.04 ± 0.007	0.02 ± 0.002	0.004 ± 0.001	BLOQ	BLOQ	BLOQ
CSF (nmol/mL)	21.99 ± 4.88	0.03 ± 0.004	BLOQ	BLOQ	BLOQ	BLOQ	BLOQ	BLOQ	BLOQ	BLOQ	BLOQ
Whole cerebellum (nmol/g)	3.86 ± 1.87	0.45 ± 0.08	0.37 ± 0.01	0.36 ± 0.02	0.37 ± 0.03	0.31 ± 0.05	0.29 ± 0.02	0.26 ± 0.06	0.35 ± 0.08	0.245 ± 0.01	0.18 ± 0.02
Left cortex (nmol/g)	4.29 ± 2.30	0.36 ± 0.02	0.32 ± 0.32	0.28 ± 0.01	0.31 ± 0.06	0.21 ± 0.02	0.17 ± 0.002	0.14 ± 0.01	0.15 ± 0.01	0.14 ± 0.05	0.16 ± 0.13
Left subcortex (nmol/g)	2.67 ± 2.17	0.37 ± 0.07	1.64 ± 2.38	0.23 ± 0.02	0.25 ± 0.04	0.12 ± 0.02	0.16 ± 0.02	0.13 ± 0.01	0.12 ± 0.01	0.15 ± 0.01	0.14 ± 0.05
Left hippocampus (nmol/g)	5.73^a^	3.52 ± 5.55	0.25 ± 0.05	0.21 ± 0.05	0.23 ± 0.02	0.15 ± 0.03	0.10 ± 0.02	0.09 ± 0.03	0.09 ± 0.01	0.12 ± 0.01	0.15 ± 0.01
Left restbrain (nmol/g)	4.27 ± 2.00	0.42 ± 0.12	0.31 ± 0.03	0.27 ± 0.004	0.35 ± 0.15	0.22 ± 0.04	0.21 ± 0.03	0.17 ± 0.02	0.13 ± 0.02	0.09 ± 0.01	0.12 ± 0.01
Skin (nmol/g)	69.37 ± 43.63	43.38 ± 41.83	9.78 ± 1.92	9.69 ± 0.78	6.89 ± 2.27	5.86 ± 2.51	3.74 ± 0.95	2.79 ± 0.86	2.26 ± 0.55	1.32 ± 0.50	1.57 ± 0.52
Right restbrain (nmol/g)	4.23 ± 1.99	0.39 ± 0.03	0.33 ± 0.04	0.28 ± 0.01	0.27 ± 0.03	0.25 ± 0.06	0.18 ± 0.003	0.15 ± 0.006	0.16 ± 0.03	0.15 ± 0.004	0.08 ± 0.008
Femur bones (nmol/g)	81.69 ± 67.46	18.46 ± 1.96	18.99 ± 0.75	21.53 ± 0.68	20.32 ± 2.33	18.05 ± 2.00	15.72 ± 0.83	13.29 ± 1.30	12.73 ± 0.87	13.35 ± 1.83	14.00 ± 0.06
Left kidney (nmol/g)	974.55 ± 82.87	296.99 ± 26.71	163.15 ± 21.51	66.24 ± 3.03	34.27 ± 9.43	11.30 ± 4.81	7.41 ± 1.18	5.98 ± 0.46	5.03 ± 1.39	5.04 ± 1.50	4.64 ± 1.21
Left liver lobe (nmol/g)	32.97 ± 5.61	32.13 ± 10.65	15.58 ± 3.08	21.40 ± 3.47	7.07 ± 2.16	5.09 ± 1.28	1.08 ± 0.40	0.67 ± 0.24	0.72 ± 0.30	1.14 ± 0.41	0.78 ± 0.25
Left lung lobe (nmol/g)	74.48 ± 28.66	10.24 ± 1.66	5.34 ± 0.55	5.34 ± 1.00	4.30 ± 1.32	3.18 ± 0.19	1.95 ± 0.64	1.05 ± 0.15	1.21 ± 0.17	0.83 ± 0.23	1.16 ± 0.25
Testes (nmol/g)	42.60 ± 17.76	4.06 ± 0.57	2.40 ± 0.07	1.63 ± 0.17	0.98 ± 0.19	0.54 ± 0.19	0.38 ± 0.09	0.45 ± 0.34	0.25 ± 0.04	0.19 ± 0.01	0.22 ± 0.04

^a^Denotes n = 1.

**Table 3:** Gadolinium concentrations as measured by ICP-MS after a single dose of gadodiamide in blood, CSF and tissues, expressed as %ID ± S.D (n = 3 per timepoint).

Tissue	Timepoint										
	1 h	1 day	3 days	1 week	2 weeks	4 weeks	6 weeks	8 weeks	10 weeks	15 weeks	20 weeks
Whole blood (%ID/mL)	4.3 × 10^–8^ ± 9.2 × 10^–10^	9.2 × 10^−11^ ± 1.0 × 10^−11^	5.8 × 10^−11^ ± 5.1 × 10^−12^	3.5 × 10^−11^ ± 1.9 × 10^−12^	2.1 × 10^−11^ ± 1.4 × 10^−12^	9.8 × 10^−12^ ± 1.0 × 10^−12^	4.8 × 10^−12^ ± 3.7 × 10^−13^	9.2 × 10^−13^ ± 2.3 × 10^−13^	BLOQ	BLOQ	BLOQ
CSF (%ID/mL)	4.7 × 10^–9^ ± 1.0 × 10^–9^	9.8 × 10^−12^ ± 1.5 × 10^−12^	2.1 × 10^−12^ ± 5.2 × 10^−12^	6.6 × 10^−12^ ± 6.6 × 10^−14^	2.3 × 10^−12^ ± 1.3 × 10^−12^	BLOQ	BLOQ	BLOQ	BLOQ	BLOQ	BLOQ
Whole cerebellum (%ID/g)	8.3 × 10^–4^ ± 4.0 × 10^–4^	1.2 × 10^–4^ ± 1.1 × 10^–5^	1.0 × 10^–4^ ± 8.5 × 10^–6^	9.5 × 10^–5^ ± 1.2 × 10^–5^	7.7 × 10^–5^ ± 9.3 × 10^–6^	6.5 × 10^–5^ ± 9.5 × 10^–6^	5.9 × 10^–5^ ± 2.5 × 10^–6^	5.8 × 10^–5^ ± 1.3 × 10^–5^	7.5 × 10^–5^ ± 1.8 × 10^–5^	5.4 × 10^–5^ ± 1.3 × 10^–6^	3.8 × 10^–5^ ± 3.8 × 10^–6^
Left cortex (%ID/g)	9.2 × 10^–4^ ± 5.0 × 10^–4^	1.0 × 10^–4^ ± 4.7 × 10^–6^	8.7 × 10^–5^ ± 1.2 × 10^–5^	7.4 × 10^–5^ ± 1.6 × 10^–6^	6.3 × 10^–5^ ± 1.1 × 10^–5^	4.3 × 10^–5^ ± 3.0 × 10^–6^	3.6 × 10^–5^ ± 1.6 × 10^–6^	3.1 × 10^–5^ ± 2.8 × 10^–6^	3.2 × 10^–5^ ± 3.4 × 10^–6^	3.0 × 10^–5^ ± 8.0 × 10^–6^	3.3 × 10^–5^ ± 2.7 × 10^–5^
Left subcortex (%ID/g)	3.4 × 10^–5^ ± 2.4 × 10^–5^	6.9 × 10^–5^ ± 6.4 × 10^–5^	4.4 × 10^–4^ ± 6.4 × 10^–4^	6.2 × 10^–5^ ± 5.0 × 10^–6^	5.3 × 10^–5^ ± 7.6 × 10^–6^	4.1 × 10^–5^ ± 3.6 × 10^–6^	3.4 × 10^–5^ ± 4.3 × 10^–6^	2.8 × 10^–5^ ± 2.7 × 10^–6^	2.5 × 10^–5^ ± 3.5 × 10^–6^	7.7 × 10^–5^ ± 4.8 × 10^–5^	6.8 × 10^–4^ ± 1.1 × 10^–3^
Left hippocampus (%ID/g)	1.1 × 10^–3^^a^	1.0 × 10^–3^ ± 1.6 × 10^–3^	6.8 × 10^–5^ ± 1.0 × 10^–5^	5.5 × 10^–5^ ± 1.6 × 10^–5^	4.5 × 10^–5^ ± 1.1 × 10^–5^	3.1 × 10^–5^ ± 7.4 × 10^–6^	2.1 × 10^–5^ ± 4.0 × 10^–6^	1.9 × 10^–5^ ± 7.1 × 10^–6^	1.8 × 10^–5^ ± 3.2 × 10^–6^	2.1 × 10^–5^ ± 9.9 × 10^–6^	1.7 × 10^–5^ ± 1.0 × 10^–5^
Left restbrain (%ID/g)	9.2 × 10^–4^ ± 4.3 × 10^–4^	1.2 × 10^–4^ ± 4.1 × 10^–5^	8.4 × 10^–5^ ± 1.2 × 10^–5^	7.2 × 10^–5^ ± 5.9 × 10^–6^	7.3 × 10^–5^ ± 3.5 × 10^–5^	4.7 × 10^–5^ ± 1.1 × 10^–5^	4.3 × 10^–5^ ± 6.8 × 10^–6^	3.8 × 10^–5^ ± 5.6 × 10^–6^	2.9 × 10^–5^ ± 4.1 × 10^–6^	3.7 × 10^–5^ ± 3.7 × 10^–6^	1.7 × 10^–5^ ± 2.3 × 10^–6^
Right restbrain (%ID/g)	9.1 × 10^–4^ ± 4.3 × 10^–4^	1.1 × 10^–4^ ± 1.1 × 10^–5^	8.9 × 10^–5^ ± 1.3 × 10^–5^	7.5 × 10^–5^ ± 4.4 × 10^–6^	5.6 × 10^–5^ ± 6.8 × 10^–6^	5.1 × 10^–5^ ± 9.9 × 10^–6^	3.7 × 10^–5^ ± 9.5 × 10^–7^	3.3 × 10^–5^ ± 9.4 × 10^–7^	3.3 × 10^–5^ ± 6.9 × 10^–6^	3.3 × 10^–5^ ± 1.3 × 10^–6^	1.8 × 10^–5^ ± 1.1 × 10^–6^
Skin (%ID/g)	1.5 × 10^–2^ ± 9.7 × 10^–3^	1.1 × 10^–2^ ± 1.0 × 10^–2^	2.6 × 10^–3^ ± 4.6 × 10^–4^	2.5 × 10^–3^ ± 2.2 × 10^–4^	1.4 × 10^–3^ ± 4.0 × 10^–4^	1.2 × 10^–3^ ± 4.6 × 10^–4^	7.7 × 10^–4^ ± 1.9 × 10^–4^	6.1 × 10^–4^ ± 1.7 × 10^–4^	4.8 × 10^–4^ ± 1.2 × 10^–4^	2.9 × 10^–4^ ± 1.2 × 10^–4^	3.4 × 10^–4^ ± 1.0 × 10^–4^
Femur bones (%ID/g)	1.7 × 10^–2^ ± 1.4 × 10^–2^	5.1 × 10^–3^ ± 5.4 × 10^–4^	5.1 × 10^–3^ ± 3.4 × 10^–4^	5.7 × 10^–3^ ± 2.6 × 10^–4^	4.2 × 10^–3^ ± 5.3 × 10^–4^	3.8 × 10^–3^ ± 1.4 × 10^–4^	3.2 × 10^–3^ ± 1.5 × 10^–4^	2.9 × 10^–3^ ± 2.0 × 10^–4^	2.7 × 10^–3^ ± 1.9 × 10^–4^	2.9 × 10^–3^ ± 5.0 × 10^–4^	3.0 × 10^–3^ ± 8.5 × 10^–5^
Left kidney (%ID/g)	2.1 × 10^−1^ ± 1.4 × 10^−2^	8.2 × 10^−2^ ± 3.0 × 10^−3^	4.4 × 10^−2^ ± 7.0 × 10^−3^	1.7 × 10^−2^ ± 4.5 × 10^−4^	7.0 × 10^−3^ ± 1.5 × 10^−3^	2.3 × 10^−3^ ± 4.0 × 10^−4^	1.5 × 10^−3^ ± 2.1 × 10^−4^	1.3 × 10^−3^ ± 1.3 × 10^−4^	1.0 × 10^−3^ ± 3.2 × 10^−4^	1.1 × 10^−3^ ± 2.8 × 10^−4^	1.0 × 10 ^−3^ ± 2.2 × 10 ^−4^
Left liver lobe (%ID/g)	7.1 × 10^–3^ ± 1.3 × 10^–3^	8.8 × 10^–3^ ± 2.4 × 10^–3^	4.1 × 10^–3^ ± 6.1 × 10^–4^	5.6 × 10^–3^ ± 1.1 × 10^–3^	1.4 × 10^–3^ ± 4.5 × 10^–4^	1.0 × 10^–3^ ± 1.0 × 10^–3^	2.2 × 10^–4^ ± 8.0 × 10^–5^	1.5 × 10^–4^ ± 5.9 × 10^–5^	1.5 × 10^–4^ ± 6.0 × 10^–5^	2.5 × 10^–4^ ± 1.0 × 10^–4^	1.7 × 10^–4^ ± 5.6 × 10^–5^
Left lung lobe (%ID/g)	1.5 × 10^−2^ ± 5.7 × 10^−3^	2.8 × 10^−3^ ± 2.1 × 10^−4^	1.4 × 10^−3^ ± 1.1 × 10^−4^	1.4 × 10^−3^ ± 1.7 × 10^−4^	8.9 × 10^−4^ ± 2.6 × 10^−4^	6.6 × 10^−4^ ± 2.2 × 10^−4^	4.0 × 10^−4^ ± 1.2 × 10^−4^	2.3 × 10^−4^ ± 3.0 × 10^−5^	2.6 × 10^−4^ ± 4.6 × 10^−5^	1.8 × 10^−4^ ± 5.5 × 10^−5^	2.5 × 10 ^−4^ ± 4.9 × 10 ^−5^
Testes (%ID/g)	9.1 × 10^–3^ ± 3.8 × 10^–3^	1.1 × 10^–3^ ± 1.5 × 10^–4^	6.4 × 10^–4^ ± 5.4 × 10^–5^	4.3 × 10^–4^ ± 3.0 × 10^–5^	2.0 × 10^–4^ ± 4.2 × 10^–5^	1.1 × 10^–4^ ± 3.3 × 10^–5^	7.9 × 10^–5^ ± 1.8 × 10^–5^	9.8 × 10^–5^ ± 7.5 × 10^–5^	5.3 × 10^–5^ ± 9.3 × 10^–6^	4.2 × 10^–5^ ± 4.2 × 10^–6^	4.7 × 10^–5^ ± 9.0 × 10^–6^

^a^denotes n = 1. Injected dose (ID).

## Discussion

Short-term persistence of gadolinium in the rodent brain after the repeated administrations of GBCAs has previously been reported as having no deleterious consequence^10,13,14^. This study characterised the long-term retention of gadolinium following repeat, high dose gadodiamide administration, and similarly reports a lack of observed toxicity in the rat brain. Given that 500 million doses of GBCAs have been administered since their introduction, it is important to understand any potential effects of these agents using realistic dosing scenarios and their clearance kinetics^19^. Previous data have shown initial rapid clearance of gadolinium followed by slower clearance long-term, matching a three-phase clearance model^11^. Initial clearance exhibits kinetic similarities that closely match serum clearance^6,20^. The second phase of clearance over a period of weeks^11^ possibly reflects delayed clearance from lymphatics or CSF^6^. This second phase has been reported in rats^11^ and mice^9^ and persistence after many serum clearance half-lives is reported with linear and macrocyclic GBCAs^9,10,14^. However, it appears that a small fraction persists longer-term without consequence, in agreement with the few clinical autopsy studies including direct gadolinium measurement^3,4,21^. Macrocyclic GBCAs exhibit faster clearance of gadolinium from tissues than linear agents and have been shown to have a lower propensity than linear agents for T1 signal intensity in the dentate nucleus and globus pallidus^5,6,14,18^.

LA-ICP-MS analysis showed diffuse gadolinium distribution in the brain with highest concentrations in the DCN. This retention with MR hyperintensity, has been observed in other studies^8,10,22^. Lower concentrations in the subcortical forebrain agree with published data^8,10^ but there is less correlation with published MR data^22^. The lack of histopathological findings in this study agrees with our previous data assessing the brain up to 20 weeks post-injection^11^ and by others at 8 weeks postdosing^8^. The lack of any histopathological findings further supports the clinical findings. A more detailed assessment facilitated by electron microscopy revealed no changes in morphology of neurons, astrocytes, oligodendrocytes, microglia, endothelium or pericytes after 1-year post dosing and gives further reassurance of biological inertness of the retained gadolinium.

In previous studies, repeated gadodiamide administration (12 mmol/kg cumulative dose) in rats resulted in retention of 0.00019% ID 1-week post-injection, and 0.00011% ID 20-weeks post-injection^11^. In this study, 20 weeks after a single gadodiamide administration the levels of gadolinium in discrete brain regions such as the hippocampus and cerebellum were lower (0.00002% ID/g and 0.00004% ID/g respectively). In the right rest brain, the gadolinium level was 0.000018% ID/g, tenfold lower than at the same time point after a cumulative dose of 12 mmol/kg (11). The higher gadolinium concentrations observed in the brain with repeat dosing suggest there is some accumulation of the injected dose with repeated administration. However, compared to the total injected dose this fraction is extremely small in either setting, with just 4/10,000,000th of the injected gadolinium dose remaining in the whole brain following a single administration and 1/1,000,000th after repeat dosing. Generally, MR hyperintensity if observed when gadolinium exceeds 1 μg gadolinium/g tissue^23^. In our study no tissue analysed demonstrated concentrations above this threshold after 3 days and thus would not result in MR hyperintensity. Many of the tissues analysed here demonstrate a time-dependant reduction in gadolinium levels, which is consistent with the elimination of gadolinium-containing gadodiamide. The small amounts of retained gadolinium may represent residual gadodiamide or gadolinium dechelated from the parent molecule^2,10^. The relatively low levels of gadolinium in the brain compared to other tissues analysed in this study is probably related to the presence of the blood brain barrier, the relatively low delivery of gadolinium into the CSF (for example through the choroid plexus) and subsequent brain distribution, compared with distribution into other organs more directly from the blood. Although Lohrke et al. demonstrated that gadolinium accumulates in the vessel basement membrane, McDonald et al. showed that in humans there is retention in the parenchyma with an intact BBB^8,24^. It is unclear how much of a contribution to hyperintensity is due to gadolinium retained in the vasculature and how much is due to gadolinium that has entered the brain parenchyma. Gadolinium has been detected in the skin (a tissue known to retain high gadolinium for all GBCAs) of rats up to 364 days post dosing^25^. Our data show an initial rapid clearance up to 4 weeks, which diminishes thereafter. This may be as a result of retention of the water insoluble gadolinium species which are less prone to elimination.

It has been proposed that GBCA stability and retention is reflected by an inverse relationship between GBCA thermodynamic stability and lability and skin retention^25^. The bone may act as a reservoir for gadolinium and could result in a chronic, slow release over time. In humans, bone retention has been detected up to 8 years after GBCA administration and is thought to be the main gadolinium reservoir in the body due to the incorporation of gadolinium by osteoblasts into the bone matrix^26,27^. Even with macrocyclic GBCAs which show lower levels of retention overall, there are sustained high levels of retained gadolinium after administration^28^. This is in line with this study, where gadolinium concentrations observed in the femur reduced rapidly 1-day post-injection (to 0.005% ID/g) with limited subsequent reduction up to 20 weeks (0.003% ID/g). Gadodiamide is excreted in the urine (> 99%) through renal elimination following first-order kinetics described by a two-compartment model of pharmacokinetics with half-lives of 3.7 (distribution) and 77.8 min (elimination) in humans^29^. In this study the kidney showed initial high gadolinium levels (0.2% ID/g) with rapid clearance up to 2 weeks (0.007% ID/g) and a moderate subsequent reduction to 20 weeks post-dosing (0.001% ID/g). This clearance of gadolinium may be a result of initial rapid kidney excretion of gadodiamide from the blood. With time and the slow release of gadodiamide from tissues into the blood, excretion may be from proximal convoluted tubules, a well-known mechanism in contrast media (27). Whilst most studies have focused on hyperintensity in the dentate nucleus and globus pallidus, the anterior pituitary has also been shown to exhibit hyperintensity in unenhanced T1-weighted images in patients with normal renal function who have had more than 5 gadodiamide administrations^30^. This signal intensity observed in the anterior pituitary which lacks a BBB, has been shown to exhibit clearance in humans over time. T1 hyperintensity is highest in the anterior pituitary MR scans of patients after a single gadodiamide administration with a minimum delay between dosing and imaging. This hyperintensity is significantly reduced after multiple gadodiamide administrations with a longer delay between administration and MR imaging. Whilst not quantitative of total gadolinium levels in the anterior pituitary, this provides evidence for clearance of GBCAs from this region in patients with normal renal function over time^31^. GBCAs may enter the brain through one or more mechanisms. Such mechanisms include diffusion across the blood CSF barrier, crossing directly through the BBB, via the perivascular (pial-glial) system or directly into regions lacking a BBB (i.e. the anterior pituitary)^6,32–36^. Blood-CSF barrier entry may occur with soluble GBCAs present in the CSF being transported through the ependymal lining and into the brain interstitium. However the majority of CSF may drain to lymph nodes and as the extracellular matrix is anionic it may impair the negatively charged GBCAs from utilizing this pathway^37^. Gadolinium foci are found in basement membranes around capillaries suggesting entry from the blood through or around the endothelia and across the BBB^3,24^. The perivascular system (pial-glial) may be involved in GBCA entry into the brain as there is evidence that molecules may be transported across the pial-glial basement membrane and are cleared through the intramural peri-arterial drainage (IPAD) located in the smooth muscle cell basement membrane, and represents a very rapid drainage pathway^37–39^. Whilst short-term clearance of gadolinium from the brain is thought to involve drainage along the basement membrane of cerebral capillaries and arterioles using the IPAD pathway, the long-term clearance mechanisms are unelucidated and require further studies to better understand long-term clearance pathways^37,40^. Using LA-ICP-MS studies to map mesoscale localization of gadolinium in the brain we see that the relative distribution of gadolinium in the brain was relatively unchanged between 1 and 50 weeks. We have previously reported measurable levels of gadolinium in the blood 1-week after dosing^11^ which is BLOQ at later time points. Even 1 week represents > 500 serum clearance half-lives and it appears that some GBCA enter a slow clearance tissue compartment, possibly the systemic lymphatics or CNS glymphatics^6^. Indeed, we and others^8^ see modest cerebro-cortical enhancement along the medial longitudinal fissure and periventricular tissue, consistent with CSF exchange in the cortex. The gadolinium that we observe in the DCN is largely vascular and on the luminal side of the BBB, which implies direct delivery via the blood. Furthermore, there is no accepted rationale for the elevated concentrations within the DCN compared to low levels throughout the brain. More research is required to resolve the potential mechanisms of brain distribution. These findings confirm that gadolinium in the brain is at trace levels, is present over long periods of time and adds to the evidence of a lack of associated tissue injury or cytologic abnormality.

Limitations of this study include an absence of 50-week timepoints for single dose gadolinium kinetics to match the repeat dose study. We did not perform ultrastructural analysis in all organs and thus whether this retention is solely within the vasculature or parenchyma of each organ would require further analysis. Although we demonstrate the absence of morphological brain changes, we acknowledge it is conceivable that gadolinium retained in the brain could have effects on neuronal function in the absence of morphological changes, even though, currently there are no data which supports this. Control animals were not included in the kinetics analysis as it is expected that no gadolinium will be present in non GBCA administered animals.

The findings of our study demonstrate that there are no further significant changes in the levels of brain gadolinium retention after 20 weeks and that there were no morphologic or ultrastructural abnormalities as well as no evidence of chronic toxicity arising from trace levels of retained gadolinium in neural tissues up to 50 weeks post administration. These results suggest that a human equivalent dose of gadodiamide results in no neural tissue damage or abnormalities and is washed out from tissues over time. These data show for the first time the kinetics of a single administration of gadodiamide in rats in a panoply of tissues and discrete brain regions, demonstrating that whilst there are varying degrees of retention in tissues, Gadolinium levels diminish up to 20 weeks after administration in the majority of tissues. This is important with regards to existing literature and future work to the understanding of different dosing regiments, the impact on retention and tissue distribution, using these more clinically relevant data as a base.
